# Switching Shiga Toxin (Stx) Type from Stx2d to Stx2a but Not Stx2c Alters Virulence of Stx-Producing *Escherichia coli* (STEC) Strain B2F1 in Streptomycin (Str)-Treated Mice

**DOI:** 10.3390/toxins13010064

**Published:** 2021-01-15

**Authors:** Beth A. McNichol, Rebecca A. Bova, Kieron Torres, Lan N. Preston, Angela R. Melton-Celsa

**Affiliations:** 1 Henry M. Jackson Foundation for the Advancement of Military Medicine, Bethesda, MD 20817, USA; beth.mcnichol01@gmail.com (B.A.M.); kieron.torres@yahoo.com (K.T.); 2 Geneva Foundation, Tacoma, WA 98402, USA; rebecca.bova.ctr@usuhs.edu; 3 Department of Microbiology & Immunology, Uniformed Services University of the Health Sciences, 4301 Jones Bridge Road, Bethesda, MD 20814, USA; 4 Multidrug-Resistant Organism Repository & Surveillance Network, Walter Reed Army Institute of Research, Silver Spring, MD 20910, USA; lan.n.preston.ctr@mail.mil

**Keywords:** Shiga toxin (Stx), Shiga toxin type 2d (Stx2d), Shiga toxin type 2c (Stx2c), STEC, B2F1

## Abstract

Shiga toxin (Stx)-producing *Escherichia coli* (STEC) strain B2F1 produces Stx type 2d, a toxin that becomes more toxic towards Vero cells in the presence of intestinal mucus. STEC that make Stx2d are more pathogenic to streptomycin (Str)-treated mice than most STEC that produce Stx2a or Stx2c. However, purified Stx2d is only 2- or 7-fold more toxic by the intraperitoneal route than Stx2a or Stx2c, respectively. We hypothesized, therefore, that the toxicity differences among Stx2a, Stx2c, and Stx2d occur at the level of delivery from the intestine. To evaluate that hypothesis, we altered the toxin type produced by *stx*_2d_+ mouse virulent O91:H21 clinical isolate B2F1 to Stx2a or Stx2c. Because B2F1 encodes two copies of *stx*_2d_, we did these studies in a derivative of B2F1 in which *stx*_2d1_ was deleted. Although the strains were equivalently virulent to the Str-treated mice at the 10^10^ dose, the B2F1 strain that produced Stx2a was attenuated relative to the ones that produced Stx2d or Stx2c when administered at 10^3^ CFU/mouse. We next compared the oral toxicities of purified Stx2a, Stx2c, and Stx2d. We found that purified Stx2d is more toxic than Stx2a or Stx2c upon oral administration at 4 µg/mouse. Taken together, these studies suggest that Stx2 toxins are most potent when delivered directly from the bacterium. Furthermore, because Stx2d and Stx2c have the identical amino acid composition in the toxin B subunit, our results indicate that the virulence difference between Stx2a and Stx2d and Stx2c resides in the B or binding subunit of the toxins.

## 1. Introduction

Shiga toxin (Stx)-producing *Escherichia coli* (STEC) are foodborne causes of bloody diarrhea, and infection may lead to a serious sequela, hemolytic uremic syndrome (HUS), which is characterized by hemolytic anemia, thrombocytopenia, and renal failure. There are two immunologically distinct groups of Stx, Stx1 and Stx2. The prototype from each group, Stx1a and Stx2a, and two other subtypes of Stx2, Stx2c, and Stx2d, are most strongly linked to HUS [1], though other Stx subtypes are occasionally associated with HUS [2]. Stx2d is unique because elastase in intestinal mucus removes the final two amino acids of the A subunit [3], a process called activation [4]. STEC that make the activatable Stx2d are more pathogenic to streptomycin (Str)-treated mice than most STEC that produce the non-activatable Stx2a or Stx2c [5,6]. However, purified Stx2d is only 2- or 7-fold more toxic to mice by the intraperitoneal (ip) route than Stx2a or Stx2c, respectively [6,7]. We hypothesized, therefore, that the toxicity differences among Stx2a, Stx2c, and Stx2d occur at the level of delivery from the intestine. In this study we switched the toxin type produced by STEC strain B2F1 from Stx2d to Stx2a or Stx2c to determine if Stx2d was the sole determinant of the high pathogenicity of B2F1 in Str-treated mice, and to evaluate if these toxins were of similar potency as delivered by an STEC from the intestine.

## 2. Results

### 2.1. Construction of Strains

**Figure 1:**
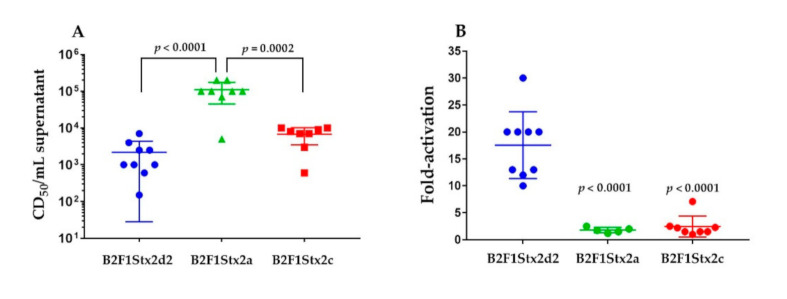
Toxicity and activation. The mean cytotoxicity of supernatants from the strains on Vero cells (**A**) and fold-activation (**B**) is shown. Fold-activation is the ratio of cytotoxicity of the supernatant after incubation with mouse intestinal mucus or the buffer control. The error bars represent standard deviation. Each point represents an individual replicate. The *p* values were determined by one-way analysis of variance (ANOVA) with Tukey’s multiple comparisons test and are relative to the B2F1Stx2a (**A**) or to the B2F1Stx2d2 values (**B**).

STEC strain B2F1 encodes two copies of *stx*_2d_. Therefore, *stx*_2d1_ was deleted from this strain since Stx2d1 is not required for B2F1 mouse virulence [8]. B2F1Δ*stx*_2d1_ was then altered through the use of suicide vector-mediated recombination to encode *stx*_2a_ or *stx*_2c_ in place of *stx*_2d2_. Table 1 shows the amino acid differences among the three toxins, and only 4 or 2 nucleotide changes were necessary to change the toxin type from Stx2d2 to Stx2a or Stx2c, respectively (see Materials and Methods for specifics). Supernatants from B2F1 that produced Stx2d2, Stx2a, or Stx2c were toxic to Vero cells as expected, Figure 1A. Furthermore, the in vitro toxicity of the supernatant from B2F1Stx2a was significantly higher than that of B2F1Stx2d and B2F1Stx2c, as predicted, due to the approximately 30- to 100-fold higher specific activity of Stx2a as compared to Stx2d2 and Stx2c for Vero cells [9]. Stx2d2 and Stx2c have essentially the same specific activity for Vero cells [9].

### 2.2. Only the Toxin from B2F1Stx2d2 Is Activatable

We next tested the toxin produced by B2F1Stx2d2, B2F1Stx2c, and B2F1Stx2a for the capacity to be activated, Figure 1B. Only the toxin from B2F1Stx2d2 was activatable, as expected, since the activity of Stx2d but not Stx2a or Stx2c is increased in the presence of mouse intestinal mucus [4].

### 2.3. B2F1Stx2a Is Attenuated

**Figure 2:**
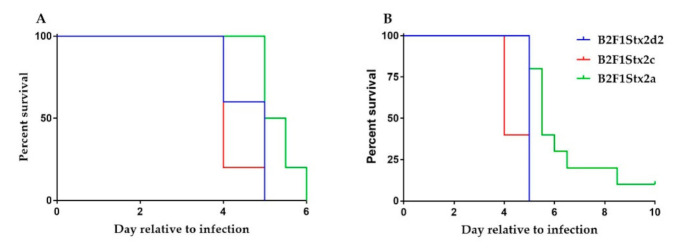
Survival curves in Str-treated BALB/c mice for B2F1Stx2d2, B2F1Stx2a, and B2F1Stx2c. (**A**) Survival after an inoculum of 10^9^ CFU/mouse. (**B**) Survival after an inoculum of 10^3^ CFU/mouse. The survival curves were not statistically different at the 10^9^ dose. At the 10^3^ dose, the B2F1Stx2a survival curve was different than both the B2F1Stx2d2 and B2F1Stx2c survival curves, *p* = 0.005 or *p* = 0.0008, respectively. *n* = 5 (B2F1Stx2d2 and B2F1Stx2c) or 10 (B2F1Stx2a) mice.

**Figure 3:**
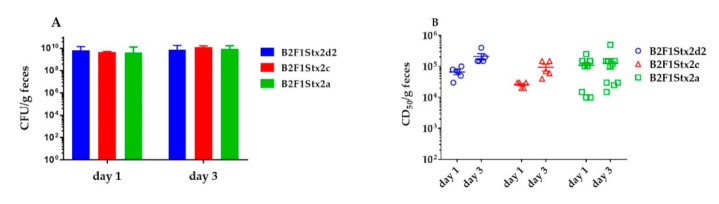
The CFU (**A**) and CD_50_/g feces (**B**) from mice inoculated with 10^3^ CFU of the indicated strains as measured on days 1 and 3 post-infection. The CD_50_/g feces were higher on day 3 as compared to day 1 for both B2F1Stx2d2 and B2F1Stx2c (*p* = 0.001), though the CFU/g feces did not change. Each symbol represents an individual mouse. *n* = 5 (B2F1Stx2d2 and B2F1Stx2c) or 10 (B2F1Stx2a) mice. Data were compared by two-way ANOVA on the log-transformed values.

We next assessed the strains for the capacity to cause a lethal infection in Str-treated mice. We found that B2F1Stx2d2 was highly lethal to mice (Figure 2) as we previously observed for a different *stx*_2d1_ mutant of B2F1 [8]. We were surprised, however, to find that at a dose of 10^9^ CFU/mouse, the survival curves were not different among the three strains. In contrast, when we evaluated a much lower dose, 10^3^ CFU/mouse, we found that B2F1Stx2a was attenuated compared to B2F1Stx2d2 and B2F1Stx2c. All three strains colonized the Str-treated mice similarly as measured by the number of CFU/g feces on days 1 and 3 (Figure 3, low dose shown). We were surprised that the level of toxin measured in the mouse stool was similar for all of the strains since Stx2a has a higher specific activity than both Stx2d and Stx2c [9] as mentioned earlier. We further found that the level of toxin detected in the feces went up from day 1 to day 3 for B2F1 that produced Stx2d2 and Stx2c, but not Stx2a (Figure 3).

### 2.4. Stx2d Is More Toxic than Stx2c & Stx2a by Oral Route

To test the hypothesis that purified Stx2d2 and Stx2c, but not Stx2a, are similarly toxic when delivered from the intestine, we gavaged each toxin by the oral route. We found that survival at the 7.5 µg/mouse dose for Stx2d, Stx2c, and Stx2a was not statistically different, though the trend was Stx2d > Stx2a > Stx2c (Table 2). However, at the 4 µg dose, Stx2d killed 5/9 mice, while Stx2a and Stx2c were not toxic. Because we found that the 7.5 µg/mouse dose of Stx2c was lethal to only 40% of the mice, we tried a higher dose of 15 µg Stx2c/mouse (*n* = 4), and found that all mice died at that dose.

## 3. Discussion

Our study suggests that Stx2d and Stx2c are equivalently toxic when produced from B2F1 in the intestines of Str-treated BALB/c mice, though when administered orally, Stx2d is more toxic than Stx2c at the 4 µg/mouse dose. Previous studies showed that Stx2c is also less toxic than Stx2d by the ip route [7]. Because earlier studies showed that purified Stx2c has a higher 50% lethal dose (LD_50_) than purified Stx2d2 [7], our results indicate that Stx2c, as delivered by B2F1 from the intestine, may be more stable than purified Stx2c given orally or by ip injection.

Additionally, our results suggest that it is the B subunit of Stx2d and Stx2c that is responsible for the lower LD_50_ of B2F1Stx2d2 and B2F1Stx2c as compared to B2F1Sx2a, since Stx2d2 and Stx2c have identical B subunits. This means that in BALB/c mice, activation of Stx2d is not required for virulence. Finally, because of our finding that mice infected with B2F1Stx2a had lower than expected levels of Stx2a in the stool, and because the Stx2d and Stx2c B subunits exhibit apparently reduced binding to Gb3 relative to Stx2a, at least in vitro [10], it may be that Stx2a binds to the intestine at greater levels, and therefore is shed into the feces at reduced levels, and perhaps trafficked to the kidney at lower levels, which would result in reduced virulence.

Finally, our results continue to show that Stx2d is more toxic than Stx2a in mice. In people both of these toxins are strongly associated with HUS [1]. However, the overall numbers of HUS patients with strains that make Stx2a is much higher. We suggest that the reasons for the higher number of HUS cases associated with Stx2a than Stx2d are likely due to (i) the smaller number of strains that carry *stx*_2d_, and (ii) the fact that most HUS-associated strains also have the locus for enterocyte effacement or LEE [1]. The LEE allows for tight association of the bacteria with epithelial cells in the intestine [11]. So far, *stx*_2d_ has only been found in association with the LEE in a relatively small number of cases [12,13].

## 4. Materials and Methods

### 4.1. Strains and Plasmids

#### 4.1.1. Construction of Derivative Strains

Strains were constructed with a combination of splice by overlap extension (SOE) PCR and suicide vector-mediated recombination, and are listed in Table 3. The laboratory strains and primers used for this study are listed in Table 4.

*B2F1*Δ*stx2d1 mutant:* First, because B2F1 has two copies of *stx*_2d_, *stx*_2d1_ was deleted. The primer pairs CKSISacI & Stx2ddelR and Stx2ddelF & AMC3RSacI were used to amplify the *stx*_2d1_ sequence with a deletion of most of the coding region and the addition of *SacI* sites at the 5′ and 3′ ends. Then those PCR products were combined with primer pair CKSISacI & Stx2ddelR and amplified again. The resultant PCR product was digested with *SacI*, ligated into pCVD442, and transformed into SY327λ*pir*. The resultant plasmid (pStx2d1del) was purified and electroporated into MFD*pir*. MFD*pir* pStx2d1del was then mixed with B2F1 and the mixture was plated on LB with ampicillin (amp) and Str to select for cointegrates. The cointegrate was then grown in LB with NaCl and sucrose as described [14] and plated on LB with amp or Str. Amp-sensitive colonies were screened by PCR and Southern blot for loss of *stx*_2d1_. The mutant strain, B2F1Δ*stx*_2d1_ (B2F1Stx2d2), was then tested for the capacity to produce Stx2d2.

#### 4.1.2. B2F1Stx2c (B2F1Δ*stx*_2d1_ *stx*_2d2 c938t,g955a_) & B2F1Stx2a (B2F1Δ*stx*_2d1_ *stx*_2d2 c938t,g955a,a1074g, c1099a_) Strains

Briefly, the *stx*_2d2_ sequence was altered by SOE PCR or by use of a Quick Change mutagenesis kit (Agilent, Santa Clara, CA, USA) such that when combined into the chromosome the resultant operon would produce Stx2c or Stx2a. A similar method for generating the change in B2F1Δ*stx*_2d1_ was followed as described for the generation of B2F1Δ*stx*_2d1_ above.

### 4.2. Sequencing and Sequence Analysis

B2F1Stx2d2, B2F1Stx2a, and B2F1Stx2c were sent to the Multidrug-resistant organism Repository and Surveillance Network (MRSN) and Walter Reed Army Institute of Research (WRAIR) for DNA extraction with a Hamilton Robot and high throughput sequencing on the Illumina MiSeq. Sequences were paired and trimmed using CLC Genomics Workbench 9. A de novo assembly was then done to create contigs. The contigs were imported into Geneious Prime and mapped to the *stx*_2d_ gene. A Mauve genome alignment was also done to look for variations throughout the rest of the genomes. The only SNPs that resulted in changes were the ones that changed the toxin types as described.

#### 4.2.1. Stx Purification

Stx2a, Stx2c, and Stx2d were purified as described previously. Briefly, lysates from strains that expressed the individual toxin were purified by immunoaffinity chromatography with polyclonal rabbit anti-Stx2 or monoclonal antibody 11E10 [17] as described [7,18]

#### 4.2.2. Vero Cell Cytotoxicity Assay

The Vero cell cytotoxicity assay was done as described previously [19]. Briefly, Vero cells (10^5^ cells/mL) were seeded into 96-well plates, then overlaid with toxin samples the following day. After 48 h of incubation, the plates were fixed in formalin, stained with crystal violet, washed with water, dried; then the absorbance read at 590 nm. The CD_50_ for each sample was the inverse of the dilution that caused 50% cell killing relative to untreated cells.

### 4.3. Mouse Studies

All mouse studies were done in accordance with the recommendations of the Guide for the Care and Use of Laboratory Animals and were approved by the Institutional Animal Care and Use Committee of the Uniformed Services University. The Str-treated mouse model was used to test virulence of these isolates as described previously [4,20]. BALB/c mice were used. For the oral toxicity study, mice were gavaged with toxin preparations as described [18].

### 4.4. Statistical Analyses

All statistical analyses were done with GraphPad Prism v7.05 for Windows (GraphPad Software, San Diego, CA, USA). Survival curves were compared with the LogRank (Mantel Cox test). All CFU or CD_50_ values were compared on log-transformed data as appropriate.

## Figures and Tables

**Table 1:** Amino acid differences among Stx2d2, Stx2a, and Stx2c.

Toxin	A Subunit Position and Amino Acid		B Subunit Position and Amino Acid	
	291	297	16	24
Stx2d2	S	E	N	A
Stx2a	F	K	D	D
Stx2c	F	K	N	A

**Table 2:** Oral toxicity of purified Stx2d, Stx2c, and Stx2a.

Toxin, Dose (µg)	Number Dead/Total	Significant p Values
Stx2c, 15	4/4	-
Stx2d, 7.5	4/5	-
Stx2c, 7.5	2/5	-
Stx2a, 7.5	6/10	-
Stx2d, 4	5/9	-
Stx2c, 4	0/10	*p* = 0.007 relative to Stx2d, 4 µg dose
Stx2a, 4	0/13	*p* = 0.002 relative to Stx2d, 4 µg dose
Stx2d, 2	0/5	-

**Table 3:** STEC strains used or constructed for this study.

Strain Name	Toxin Genes Encoded	Toxin Encoded	Reference
B2F1	*stx*_2d1_ & *stx*_2d2_	Stx2d1 & Stx2d2	[15]
B2F1Stx2d2	*stx*_2d2_	Stx2d2	This study
B2F1Stx2a	*stx*_2d2 c938t, g955a, a1074g, c1099a_	Stx2a	This study
B2F1Stx2c	*stx*_2d2 c938t, g955a_	Stx2c	This study

**Table 4:** Laboratory strains, plasmids and primers used in this study.

Strain, Plasmid or Primer	Characteristics or Sequence	Reference
SY327λ*pir*	Supports replication of pCVD442	[14]
MFD*pir*	Supports replication of pCVD442; used for mating	[16]
pCVD442	Suicide plasmid; amp resistance	[14]
pSQ343	*stx*_2d1_	[9]
pSQ543	*stx*_2d2_	[9]
pMJC1	*stx*_2d2_ c938t (S291F)	This study
pKMT15	*stx*_2d2_ c938t g955a (S291F, E297K)	This study
pKMT16 *	*stx*_2d2_ a1074g, c1099a (N16D, A24D)	This study
pKMT17 *	*stx*_2d2_ g955a, a1074g, c1099a (E297K, N16D, A24D)	This study
pKMT18 *	*stx*_2d2_ c938t, g955a, a1074g, c1099a (S291F, E297K, N16D, A24D)	This study
CKS1SacI	CTTTAGCTCAGTGGTGAGAGCTCGCGACTCATAAT	This study
Stx2ddelR	CCGCCGCCATTGCATTAACAGATACAGGTGTTCCTTTTGGC	This study
Stx2ddelF	GCCAAAAGGAACACCTGTATCTGTTAATGCAATGGCGGCGG	This study
AMC3RSacI	GCCTCCCGGTGAGCTCAGTCCGGTG	This study

* These clones start at nucleotide 380 relative to the “a” in the first atg of the *stx*_2d2_ sequence.

## Data Availability

Not applicable.
